# ‘They never mentioned this in medical school!’ A qualitative analysis of medical students’ reflective writings from general practice

**DOI:** 10.1080/02813432.2023.2263486

**Published:** 2023-11-29

**Authors:** Bente Prytz Mjølstad, Linn Okkenhaug Getz

**Affiliations:** a General Practice Research Unit, Department of Public Health and Nursing, Faculty of Medicine and Health Sciences, Norwegian University of Science and Technology, NTNU, Trondheim, Norway; b Saksvik Medical Center, Hundhammeren, Norway

**Keywords:** General practice, medical students’ views, medical education, primary care curriculum, reflective writings, uncertainty, complexity

## Abstract

**Objective:**

The aim of the study was to identify final-year medical students’ experiences with thought-provoking and challenging situations in general practice.

**Design setting and subjects:**

We conducted a qualitative analysis of 90 reflective essays written by one cohort of Norwegian final-year medical students during their internship in general practice in 2017. The students were asked to reflect upon a clinical encounter in general practice that had made a strong impression on them. A primary thematic content analysis was performed, followed by a secondary analysis of encounters that stood out as particularly challenging.

**Main outcome measures:**

Clinical scenarios in general practice that make students feel professionally ‘caught off guard’.

**Results:**

The analysis identified several themes of challenging student experiences. One of these was ‘disorienting encounters’ for which the students felt totally unprepared in the sense that they did not know how to think and act. Five different scenarios were identified: (1) patients with highly distracting appearances, (2) ‘ordinary consultations’ that suddenly took a dramatic turn, (3) patients who appeared unexpectedly confrontational or devaluating, (4) scornful rejection of the young doctor’s advice, and finally, (5) confusion related to massive contextual complexity.

**Conclusions:**

Disorienting encounters stood out as particularly challenging clinical experiences for medical students in general practice. These scenarios evoked an acute feeling of incapacitation: not knowing what to think and do. Further curriculum development will focus on preparing the students to ‘know what to do when they don’t know what to do’.

## KEY POINTS

- Final-year medical students experience several challenging clinical scenarios in general practice, for which the curriculum has prepared them at least to a certain extent.
-However, scenarios occasionally occur, for which students feel totally unprepared. In such ‘disorienting’ encounters they experience moments of not knowing what to do that conflict with their ideas of what it means to be a professional.
-The undergraduate curriculum in general practice should aim to prepare students for unexpected, bewildering scenarios to prevent experiences that could alienate the students from general practice as a potential career choice.

## Background

### Primary care as a learning arena in the undergraduate curriculum

In primary care, there is open access to seek a general practitioner (GP) for a wide range of reasons and undifferentiated presentations of illness. In this generalist setting, complex problems are the rule rather than the exception. Working with complexity is advanced medical work and one of the GPs’ areas of expertise [1,2]. As part of this, GPs encounter the interconnectedness between life experiences, social living conditions and health [1–3]. Consequently, a good GP is not only patient-centred in a communicative sense but also attentive to the whole person-in-context (Nordic Federation of General Practice’s core values) [4]. Over time we have worked to develop the general practice curriculum at the Norwegian University of Science and Technology (NTNU) to prepare our students better for the lived realities in primary care. In addition to the above-mentioned characteristics of the discipline, we put increasing emphasis on how adverse life experiences and neglect can impact on a person’s capacity to engage in trusting and constructive relationships, including encounters with healthcare personnel [5,6].

Through clinical internship periods, medical students are given an opportunity to practice important skills and learn, both directly from their own experiences and from their tutors as role models [7,8]. Medical students’ reported challenges in this setting vary, depending on both the level (year of study) and clinical context. Their reflections regarding internships in hospitals (internal medicine, surgery, psychiatry) typically revolve around challenging communication and interaction with patients and relatives [9–12]. Research on internships in primary care has shown how medical students can be ‘grappling’ with different emotions as they deal with the complexity of challenging clinical encounters, leaving them overwhelmed while experiencing a gap between theory and practice [13,14].

The ability to recognise and use one’s own feelings constructively in the context of authentic clinical work can pave the way to important learning and facilitate the development of resilient professionals [15]. Primary health care represents a highly relevant arena where this can happen. Students can actively engage and take responsibility in a variety of patient encounters, gradually developing their professional identity and robustness [16]. In line with this, Regulations on the national guideline for medical education, recommends longer periods of placement in primary care for medical students [17].

Among medical students in Norway, at least one-third should ideally be motivated for a career in general practice to meet the needs of society [18]. However, Norway, like many other countries, face difficulties recruiting and retaining GPs. The are several reasons for this, but one fundamental factor to counteract this trend is to offer medical students a rewarding and memorable learning experience in general practice, motivating them to ‘care for people where they live’ [19,20]. To reach this goal, we aim to offer stimulating, interactive learning activities that can subsequently also represent a resource for curriculum development [7].

### Reflection on clinical experience—an important element of undergraduate training

In the undergraduate education of medical students, there is an increased focus on active learning processes, including reflection in practice. Following in the wake of educationalists such as John Dewey and Donald Schön, substantial literature exists in the field [15, 21–25]. The International Association For Medical Education (AMEE) defines reflection as ‘a metacognitive process that occurs before, during, and after situations with the purpose of developing a greater understanding of both the self and the situation so that future encounters with the situation are informed from previous encounters’ [8]. Mann et al. conducted a systematic literature review of the use of reflective practices in healthcare in 2009. They found that reflections carried out by health professionals have several functions, including helping understand complex situations and to learn from experience. The anticipation of challenging situations also seems to stimulate reflection. There seem to be individual differences in the ability to reflect, but this ability can be enhanced in a stimulating learning environment in which good mentors and supervisors are crucial [22]. Various examples of the use of reflection in medical education exist. Schei et al. describe how reflection can be used in teaching medical students to promote understanding of living with a serious illness [25]. Bennett et al. have documented a mismatch between practice and the formal curriculum regarding medical students’ perspectives of professional roles [26].

### Reflection task for final-year medical students in general practice at NTNU

**Figure 1.**
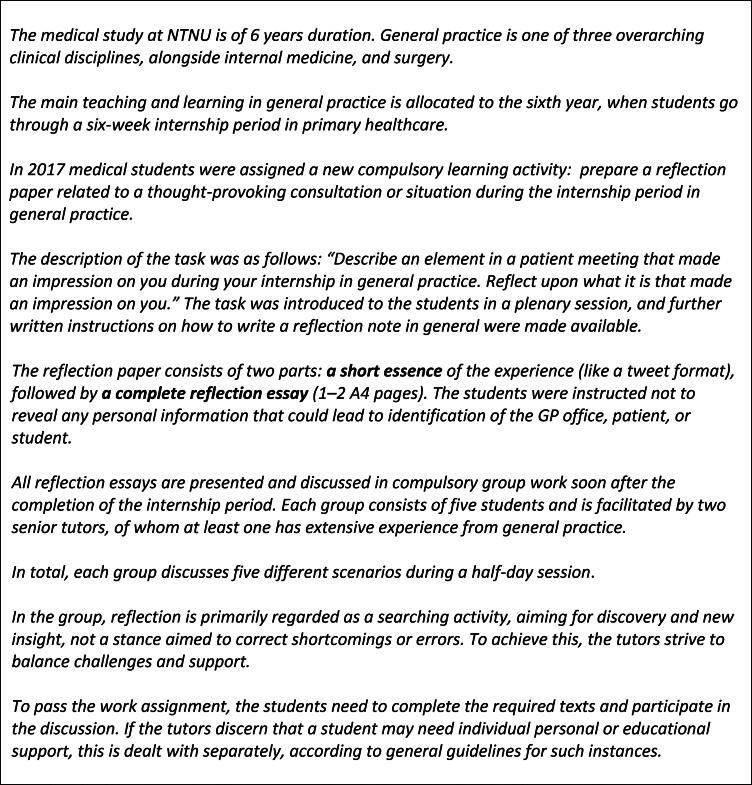
Reflective writing task and group discussion as part of teaching in general practice at NTNU.

In 2017, we added a new learning activity to the curriculum in general practice as we introduced reflective writing and a subsequent group discussion for our final-year students. In short, the students are asked to reflect upon a particularly memorable and thought-provoking experience associated with a clinical encounter in general practice during their internship period (see Figure 1).

Our motivation for the new learning activity was twofold. As part of the university’s curriculum in professionalism, a broad aim is to encourage and train students to reflect upon and discuss challenging experiences in an open and constructive manner [27]. Another aim for us as teachers in general practice has been to familiarise ourselves more with situations and scenarios in practice that have a strong impact on the students. At worst, these experiences may alienate them from general practice as a potential career choice. Giving the students the task of identifying, describing, reflecting upon, and discussing such situations represents one way to identify phenomena that could be highlighted in our teaching as part of preparing the students for the realities of clinical practice.

### Objective of this study

The aim of this study was to identify final-year medical students’ experiences with challenging situations in general practice by analysing reflective notes on memorable clinical encounters.

## Materials and methods

### Study material

This study is based on writings from the first cohort of 90 final-year medical students who wrote reflection essays based on their experiences during a six-week internship in general practice in September–November 2017 (see Figure 1 for a description of the pedagogical setting for the reflections). The reflection essays were submitted as paper copies, devoid of any personal information that could identify the GP office, patients, or themselves as students, including their own gender. The written material was thus anonymous. During the analysis of the material, the identity of the participants was not known to the researchers.

### Data analysis

**Figure 2.**
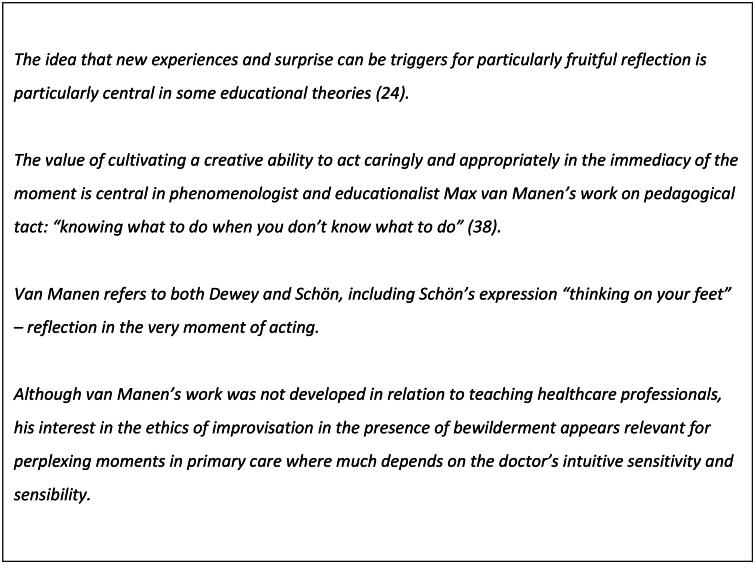
‘Knowing what to do when you don’t know what to do’.

Reflective writing can be assessed based on the content or depth of reflection. Based on the purpose of this study, we chose to analyse the content using Braun and Clarke’s reflexive thematic analysis searching inductively for themes across the data material [28,29]. The method is theoretically flexible, opening for guidance by different approaches. Experiential learning theory was the point of departure for our study. Learning from experience builds on constructivism; the student is considered an active co-creator of knowledge, and learning is the result of a process where knowledge is created through the transformation of experience [27]. Our analysis of the reflection notes followed the steps described by Braun and Clark. In the primary analysis the authors (BPM and LG) familiarised themselves with the data, reading all the reflective essays separately and making individually observational notes. In the following steps the identified themes were reviewed collaboratively in meetings as we compared our findings and subsequently condensed them into major themes. Possible overlapping themes were discussed and reviewed. Twelve distinctive thematic categories of challenging scenarios in general practice were identified (see supplementary 1). From our perspective as GP teachers, eleven of the categories covered recognisable challenges that are addressed to some extent in the existing curriculum. The latter category, however, stood out as different and caught our attention. It represented a collection of encounters where the students described how they felt utterly ‘lost’; unprepared, bewildered and personally exposed in an unpredicted and disturbing manner. We chose to submit this category of encounters to further (secondary) analysis. As the last part of the analysis, we revisited the entire data material to ensure that the chosen scenarios were the most relevant within this ‘caught off guard’ category. To strengthen the credibility and confirmability of the results, our findings were also discussed with peer researchers experienced in tutoring medical students. In the final step, the writing-up, the results were considered in the light of theoretical models and learning theories; threshold concepts, psychological safety and, finally, van Manen’s concept pedagogical tact (see Discussion including Figure 2).

### Ethics

Collection of the pedagogical material did not fall within the mandate of the Norwegian Health Research Act, thus no formal applications to the Regional Ethical Committee or the Norwegian Centre for Research Data were required. The study was approved by NTNU as an academic curriculum development program, and informed consent was obtained from all participants to the use of reflection papers for local curriculum development and scientific analysis/publication.

## Results

In our primary analysis of the 90 reflection essays, several categories of challenging situations in general practice were identified. These included difficulties identifying themselves with the patients, facing own prejudices, or facing clinical uncertainty as a professional. Some of the students had for the first time encountered the lived realities of suffering and socioeconomic deprivation in a clinical setting. Others described problems tackling complex health problems and sensitive, taboo topics. Some wrote about dilemmas in relation to the gatekeeper role and harm rising from medical (over)activity. In addition, came a variety of communication challenges, e.g. language barriers, late arriving agendas in the consultation and dealing with demanding patients or relatives. While real-life encounters with these scenarios in the role as attending physician made a strong impression, the students should be able to relate their experiences to existing curriculum content [5,6]. As teachers, we do not believe these categories point to overt pedagogical ‘gaps’ in our curriculum, although the students might obviously profit from being better prepared for these situations.

The final analytic category, in contrast, stood out as different. It assembled reflections on encounters where the student, in the role as attending physician, experienced overwhelming bewilderment and disorientation, feeling ‘caught off guard’. The reflective essays in this category, thematically named ‘disorienting encounters’, encompassed five different scenarios: (1) patients with highly distracting appearance or behaviour; (2) encounters with abrupt and unexpected turns in the consultation; (3) patients or relatives perceived as unreasonably callous and confrontational; (4) patients who refused to be examined or follow advice; and (5) encounters with unruly complex issues that evoked a feeling of chaos and professional disempowerment. In the following, we present illustrating examples for each of these five scenarios.

### Different scenarios of disorienting encounters

#### Encounters with patients with highly distracting appearance or behaviour

Some students had reflected on how the remarkable appearance or behaviour of a patient made them personally insecure in an unexpected and uncomfortable manner. This could be related to strange behaviour, a fearsome/scary appearance, an involuntary comic situation, age-inappropriate behaviour, or unspoken discrepancy between how the patient looked and what was said or expressed.

One student described how s/he felt flabbergasted and speechless after discovering that the next patient, a young person with chronic pain, wore a strikingly strange homemade blindfold. It was made of a scarf that was taped around the head of the patient, looking rather bizarre:

I wasn’t prepared to communicate with the patient without being able to make eye contact. I was also a bit put off. What made the patient choose that badly handmade blindfold? Why not just wear sunglasses? (Student 6)

Another student explained how s/he was ‘put off’ by a patient with a striking haircut and clothing who insisted to remain standing during the entire consultation for no apparent reason despite repeated invitations to sit down. At one point, the patient also got behind the student, uncomfortably close, trying to read what s/he wrote on the computer. The student described how the entire consultation had been coloured by how unsure s/he felt in that situation:

I was astonished by the intensity of my reaction to the patient’s body language. I felt disoriented and had to try to work out where to place myself. (Student 52)

#### Encounters with an abrupt and unexpected turn

Some of the students described clinical encounters where they at some point became unexpectedly overwhelmed by their own feelings. The students described being ‘taken aback’ by an abrupt turn in what they, until then, had perceived as ‘an ordinary, straightforward’ consultation. It could be related to unexpected clinical signs of serious or even fatal illness or difficult medical dilemmas.

One of the students described a situation of talking calmly with a young patient who verbally presented a seemingly banal problem, only to be ‘put off balance’ as the patient suddenly removed her scarf and revealed a tumour so large that the trachea was displaced to the opposite side.

I was astonished by the discovery. The thought of serious illness was inevitable, and this put me ‘off balance.’ (Student 56)

Another student wrote about a situation where s/he ‘stumbled into’ a difficult medical dilemma. A patient with a history of substance abuse asked the student to prescribe sedative medication to help him cope with his grief after losing his child. On the one hand, the student felt immense sympathy for the patient, while on the other hand, s/he presumed it would be considered medically wrong to prescribe addictive medication in light of the patient’s history of previous substance abuse. The student was ‘caught between empathy and guidelines’ and began to doubt his/her own judgement and entitlement to exert professional power:

And who am I, really? A simple medical student, sitting here, denying a heartbroken human being who has just lost his daughter something he sincerely requests. Can I do that? (Student 8)

#### Encounters with patients or relatives perceived as unreasonably confrontational

Several students wrote about encounters where they experienced the patient behaving callously confrontationally from the outset of the encounter, expressing irritation, anger, or overt aggression. In some instances, the student felt personally degraded, either by the patient or by an accompanying relative.

One student described a meeting with an adolescent accompanied by his/her mother, seeking medical advice for a stomach-ache. After a thorough anamnesis and examination, the student concluded that serious disease could be ‘ruled out’ but could not immediately give a precise diagnosis. The student tried his/her best to reassure the patient whilst being open about the elements of uncertainty. Nevertheless, the student experienced that the patient’s mother sneered at the doctor’s proposed solution, leaving the student disempowered:

I ended up feeling so belittled, I went to fetch my supervisor. In fact, even though my supervisor didn’t say much more than I had already tried to convey myself, I felt his statements had more impact. (Student 59)

Another patient consulted with mental health problems. Initially, he/she appeared to be trembling and anxious, and the student tried to be empathetic. Suddenly, the student experienced how the tone in the consultation changed. The student described it as a turning point when the patient without warning confronted him/her in a way that felt like a ‘reprimand’.

I had repeatedly said ‘I understand’ during the conversation, and suddenly, the patient confronted me by asking, ‘Exactly what is it, actually, that you have understood?’

(Student 41)

The student later learned that the patient had several bad experiences with so-called ‘understanding’ health personnel in his/her childhood. He/she was provoked by health personnel saying that they understood his/her situation when everything indicated that they did not.

#### Encounters with patients who refused to be examined or follow advice

Some students described their first meeting with patients who did not want to do as the doctor suggested. For example, some patients were unable or did not want, and some even explicitly refused, to be physically examined. Others were explicitly unwilling to follow medical advice. This created uncertainty and a feeling of failure, not being able to do the job ‘professionally’, as illustrated in the following examples.

One student described a middle-aged patient with Down syndrome who refused to have his/her ears examined despite the student’s many attempts to establish a safe climate. This left the student with an experience of disempowerment in dealing with even the simplest medical problem:

I was very frustrated to have failed in my attempts at examination and had not been able to clarify the problem and rule out a potentially more serious cause. At the same time, it was too discomfortable to violate the patient’s right to self-determination. (Student 33)

Another student described an encounter with a patient with several chronic serious illnesses and how s/he was surprised by the patient’s sudden confession that s/he had never taken any of his/her prescribed medications. The student described the patient as a resourceful person with more faith in alternative medicine. The student was unsure of the patient’s motivation to suddenly reveal this to her/him. Did the patient just want to provoke the student, or did s/he actually share the information on the basis of genuine trust?

The patient’s statement came out of the ‘blue’, even though s/he already had sent me several strange glances, almost like s/he had a secret s/he was about to reveal. I was relatively put out and could not come up with any good response. (Student 28)

#### Encounters with unruly complex issues that evoked a feeling of professional disempowerment

Several students had felt overwhelmed in consultations they described as complex or even chaotic. The scenarios varied and ranged from ‘simple’ clinical scenarios embedded in highly complex communicative patterns, to more dramatic situations where much was at stake.

A patient consulted for common and relatively mild acne. The student, however, immediately became caught by the possibility of significant, underlying disease, i.e. polycystic ovarian syndrome (PCOS). Without it being the intention, the student experienced the conversation going onto the wrong track, and the questions that were supposed to be clarifying turned out to be confusing, initiating anxiety and worries in the patient:

Once we had embarked on this ‘hypothetical journey’, it was difficult to turn back.

I became entangled in an explanation that I could not get out of. (Student 44)

It turned out that the reason for this approach was that the student had been affected by the fact that earlier in the day, s/he had met a patient who had a very rare condition, thereby having become sensitised in relation to looking for grave diagnoses.

A patient of foreign origin consulted the doctor for blood coming from her ear, bringing an underage child with her. The child displayed unruly behaviour, running around the room, shouting and throwing objects. The student noted that the mother almost seemed to ignore the child. In this chaotic setting, it emerged that the woman was a victim of domestic violence. The student struggled to maintain focus on the conversation whilst beginning to wonder if the child’s behaviour might be linked to the mother being exposed to violence. The student was unable to ‘take control over the situation’ and ended up thinking ‘this just doesn’t work’. In the reflection essay, s/he noted,

They never mentioned this in medical school, about the complexity. (Student 22)

## Discussion

### Main findings

A thematic analysis of 90 reflection essays from final-year medical students who described memorable and thought-provoking experiences from their clinical internship in general practice, led to the identification of several challenging situations. Most of these depicted situations for which our university’s formal curriculum is likely to have prepared the students to some extent, i.e. the experiences appear to have expanded the students’ understanding of a challenging but recognizable topic. In contrast, one of the thematic categories stood out as different. It encompassed disorienting experiences for which the students felt totally unprepared. In a sense, they were brought beyond their horizon of professional imagination. The scenarios evoked an acute feeling of incapacitation: not knowing what to think and do. Further analysis of these ‘disorientation’ encounters gave rise to five different scenarios, as previously described.

### Strengths and limitations

A major strength of this study is that it includes reflection essays from a complete cohort of final-year medical students at our university. The data represent a broad range of reflections on various practice experiences from different GPs’ offices in both rural and urban areas. The task was open-ended in the sense that it was prompted by a simple instruction to reflect on a consultation that ‘made an impression on you’ or was ‘thought-provoking’. Beyond technical instructions, no examples were given. The writing itself took place in advance of the group evaluation so that each student worked independently in the reflective process.

The reflective notes were analysed by BPM and LG, who both have extensive experience from student teaching at the sixth-year level. BPM is a specialist in general practice, a contract GP, and an associate professor of general practice. LG is a medical doctor with broad clinical experience, a professor of behavioural sciences in medicine, and the head of the General Practice Research Unit at NTNU. Both are involved in local medical education development.

We are aware that the relatively open-ended task to present ‘an experience that had made an impression’ represents a relatively unfamiliar type of educational challenge in our curriculum. The fact that the students knew they were going to present their reflections in a group setting may have influenced their choice of experience to document and share in their peer group. However, the selected scenarios can be seen as valid in the sense that they appeared to be recognisable, interesting, and meaningful both to us as researchers and to the experienced GPs who tutored the group discussions. Furthermore, the reflection notes and analytical categories presented in this paper do not differ systematically or significantly from what has been presented and discussed in subsequent student cohorts where both authors have remained active as tutors. It is also relevant to note that completion of the work task was compulsory, whilst the qualitative content of each student’s reflections was not graded as in a pass/fail exam. The reflective notes included in this study stem from one student cohort at a given university in Norway. In comparable but nevertheless different educational contexts, reflective notes might take on another character.

### Findings in relation to comparable studies

Seen together, the thematic categories identified by the primary analysis cover recognisable challenges associated with the clinical complexity of general practice [2,3]. The topics were associated with communication, the doctor–patient relationship, complex medical issues, and sensitive and taboo topics. The students also reflected on encounters with suffering and socioeconomic deprivation as well as various aspects of insecurity associated with being an inexperienced clinician. Here, we see thematic similarities with the previously mentioned U.S. study from family practice clerkship [13] and also with a Swedish study of final-year medical students’ reflective accounts about memorable consultations from their internship in primary care [14].

### The bewilderment challenge: ‘Knowing what to do when you don’t know what to do’

A common characteristic of the ‘disorienting encounters’ subjected to the secondary and closer analysis was that no guiding manual or blueprint was at hand for the students in the role as inexperienced professionals-to-be. Without warning, they simply had to rely on their personal, intuitive sensibility and sensitivity, and this seemed to render them disempowered and vulnerable. To some extent, this resonates with a subgroup of accounts in the previously mentioned study from American family practice clerkship, where some students felt overwhelmed as they grappled with lived reality and experienced a gap between theory and practice [13].

To most GPs, the disorienting scenarios will probably be recognised as peculiar and somewhat challenging from a communicative perspective. Over time, however, a GP can expect to mature and develop a *cognitive readiness for unexpected scenarios,* a professional ability to ‘see the big picture’ and extrapolate from previous experience to new, unfamiliar situations [30]. ‘Becoming familiar with the unfamiliar’ can potentially be viewed in light of *threshold concepts* theory, which describes how learners, through pondering and struggle, overcome barriers to understanding and handling their specific discipline/profession [31–33].

### Implications for curriculum development in general practice

As previously noted, most of the categories of memorable encounters resulting from this study addressed challenging situations for which the formal curriculum is likely to have prepared the students to some extent. In these cases, we conclude that the opportunity for ‘reflection in practice’ [23, 27] contributes to the envisioned learning outcome.

Based on our new insight, we are motivated to stimulate medical students’ readiness for and capacity to cope with clinical situations where they feel bewildered and vulnerable, not knowing what to do, in a manner that evokes fear of ‘losing face’. This aligns well with an increasing interest in *psychological safety* in the context of medical education [34,35]. Experiences which evoke personal insecurity and vulnerability may have a significant impact on the learner, for better or worse. As a profession, medicine has deep roots in *a culture of honour*, with a learning environment that can be punitive and intolerant of uncertainty and indecisiveness [34, 36,37]. The more unprepared the students are for moments of uncomfortable bewilderment, the more likely it seems that such experiences might hurt their self-esteem and lead to self-blame and avoidance, as opposed to a spirit of inquiry with a potential for personal and professional maturation [35,36, 38]. With reference to educationalist Max van Manen, we are thereby motivated to see if we can better prepare students to ‘know what to do when they don’t know what to do’ [38] (see Figure 2).

This entails preparing students for situations where they will feel ‘caught off guard’ so that they can assign such experiences to a pattern that represents a common challenge for young professionals, as opposed to individual experiences of failure and self-blame. Furthermore, we will invite students to discuss how they, as potential patients, would prefer an unexperienced young doctor to react and offer some practical advice for handling such situations: stay calm and do nothing hasty; be present as a fellow human being; reflect your surprise or bewilderment in a gentle and thoughtful manner, without blaming the patient.

## Conclusion

Final-year medical students at our university appear reasonably well prepared for most scenarios that make a strong impression on them during their six-week internship in clinical general practice. Nevertheless, we see room for improvement; students can be better prepared for situations where they feel professionally ‘caught off guard’, bewildered, and vulnerable. Further curriculum development will aim to help students recognise and assign disorienting encounters to a recognisable category of experiences that may stimulate and strengthen them, as opposed to upset and silence them, as young medical professionals.

## Supplementary Material

Supplemental Material
