# Supplementary material for: ‘They never mentioned this in medical school!’ A qualitative analysis of medical students’ reflective writings from general practice
Source: Scand J Prim Health Care. 2023 Nov 29;41(4):417–26. doi: 10.1080/02813432.2023.2263486 (PMC11001360; doi:10.1080/02813432.2023.2263486)
Supplement: Supplemental Material [file IPRI_A_2263486_SM9723.docx]

### Supplementary material

S 1. Overview of the 12 thematic categories of particularly memorable and thought-provoking experiences identified in the primary thematic analysis of the complete material of 90 reflection essays with illustrative quotes.

| Number | Category (main themes) | Example (quotes) |
| --- | --- | --- |
| 1 | **Disorienting encounters (being caught “off guard”)** | *“They never mentioned this in medical school – about the complexity”* |
| 2 | **Facing the lived realities of suffering and socioeconomic deprivation** | *“What do I know, being so young?” “It’s a new world”* |
| 3 | **Identification with patient** | *“It could have been me . . .”* |
| 4 | **Facing one’s own prejudices** | *“I was, even before the meeting with the patient, closed in my mindset”* |
| 5 | **Tackling a complex health problem** | *“For every symptom I asked about, the patient mentioned new symptoms”* |
| 6 | **Dilemmas related to the GP’s gatekeeping role** | *“How can I say no to the patient?”* |
| 7 | **Tackling overt “orders” from demanding patients** | *“The distribution of roles in the room was challenged, and I lost control”* |
| 8 | **Dilemmas and harm arising from medical activity** | *“For the first time, I have met a patient whom ‘we as doctors’ have made ‘sick’. It created a lot of concern for the patient”* |
| 9 | **Communication challenges and language barriers** | *“It was difficult to formulate the questions in a neutral and non-offensive way”* |
| 10 | **Tackling sensitive and taboo topics** | *“I got the impression that the patient’s view of the consultation was that it was a burden rather than support”* |
| 11 | **The impact of latent agendas on the consultation** | *“What is the patient really coming for? What is hidden beneath the surface?”* |
| 12 | **Facing doubt and uncertainty as a professional** | *“What if I am wrong?”* |
